# The Journey of Zanzibar’s Digitally Enabled Community Health Program to National Scale: Implementation Report

**DOI:** 10.2196/48097

**Published:** 2023-10-09

**Authors:** Erica Layer, Salim Slim, Issa Mussa, Abdul-Wahid Al-Mafazy, Giulia V R Besana, Mwinyi Msellem, Isabel Fulcher, Heiko Hornung, Riccardo Lampariello

**Affiliations:** 1 D-tree International Norwell, MA United States; 2 Ministry of Health Zanzibar United Republic of Tanzania; 3 D-tree International Zanzibar United Republic of Tanzania; 4 Office of the Chief Government Statistician Zanzibar United Republic of Tanzania; 5 Ministry of Health Public Health Laboratory Zanzibar United Republic of Tanzania; 6 D-tree International Lusaka Zambia

**Keywords:** Zanzibar, digital health, community health, health systems strengthening, maternal health, child health, data for decision-making, implementation science, health systems, healthcare infrastructure, health care, implementation report

## Abstract

**Background:**

While high-quality primary health care services can meet 80%-90% of health needs over a person’s lifetime, this potential is severely hindered in many low-resource countries by a constrained health care system. There is a growing consensus that effectively designed, resourced, and managed community health worker programs are a critical component of a well-functioning primary health system, and digital technology is recognized as an important enabler of health systems transformation.

**Objective:**

In this implementation report, we describe the design and rollout of Zanzibar’s national, digitally enabled community health program–Jamii ni Afya.

**Methods:**

Since 2010, D-tree International has partnered with the Ministry of Health Zanzibar to pilot and generate evidence for a digitally enabled community health program, which was formally adopted and scaled nationally by the government in 2018. Community health workers use a mobile app that guides service delivery and data collection for home-based health services, resulting in comprehensive service delivery, access to real-time data, efficient management of resources, and continuous quality improvement.

**Results:**

The Zanzibar government has documented increases in the delivery of health facilities among pregnant women and reductions in stunting among children younger than 5 years since the community health program has scaled. Key success factors included starting with the health challenge and local context rather than the technology, usage of data for decision-making, and extensive collaboration with local and global partners and funders. Lessons learned include the significant time it takes to scale and institutionalize a digital health systems innovation due to the time to generate evidence, change opinions, and build capacity.

**Conclusions:**

Jamii ni Afya represents one of the world’s first examples of a nationally scaled digitally enabled community health program. This implementation report outlines key successes and lessons learned, which may have applicability to other governments and partners working to sustainably strengthen primary health systems.

## Introduction

### Background

The World Health Organization estimates that high-quality primary health care services can meet 80%-90% of health needs over a person’s lifetime [1]. However, in many low-resource countries, this potential is severely hindered by a constrained health care system tackling significant shortages of health workers and the stock of lifesaving medical supplies, all of which limit progress toward universal health coverage (UHC) and damage trust among users [2,3]. There is a growing consensus that effectively designed and managed community health worker (CHW) programs are a critical component of a well-functioning primary health system [4-6]. CHWs have significant potential to extend health services to people’s homes, build relationships and meet individual health needs, improve access to services, and improve health system performance [7]. In addition, CHW programs have been shown to deliver a 10:1 return on investment due to increased productivity from a healthier population [8]. Despite this potential, scale-up and management of CHW programs has been slow and often underprioritized by governments [9].

Digital technology has been increasingly recognized as a critical tool to improve both access to health care and the quality of health care delivery, even for CHW programs [10]. Mobile apps, which are designed to fit the local context and are based on program guidelines, can support CHWs to register households and individuals in their communities, thus guiding CHWs through home-based visits following government guidelines. These tools, when integrated into broader digital and public health systems, can improve supervision, coordination, and program monitoring through access to real-time client-level data.

### Zanzibar’s Community Health Context

The islands of Zanzibar are a semiautonomous region of Tanzania with a population of 1.9 million people. One in 33 women in Zanzibar are estimated to die of maternal complications in her lifetime [11], 20% of children are stunted [12], and Zanzibaris face a high burden of noncommunicable diseases [13]. There is a significant human resource shortage that exacerbates health system challenges; currently, Zanzibar is lacking more than 3900 health workers, which implies a 35% shortage. A key priority for the Ministry of Health Zanzibar is to strengthen its primary health system, including community health [14].

Until recently, community health programs in Zanzibar were traditionally siloed by vertical health programs, funded by donors, and implemented by nongovernmental organizations (NGOs) with programs ending based on donor funding cycles and thematic areas of interest. This resulted in lack of coordination, duplication of efforts, and hindered long-term sustainability. In 2018, the Revolutionary Government of Zanzibar (RGoZ) took the pivotal step of formalizing a government-led community health program, bringing together all previously siloed, donor-funded programs under a single national program, making the pivotal decision to digitize the community health workforce from the start.

In this implementation report, we describe the design and rollout of Zanzibar’s national digitally enabled community health program. Our aim is to share lessons learned, drivers for success, and best practices to help other countries succeed in designing, scaling, and sustaining digitally enabled community health programs. This implementation report follows the iCHECK-DH (Guidelines and Checklist for the Reporting on Digital Health Implementations) [15].

## Methods

**Figure 1:**
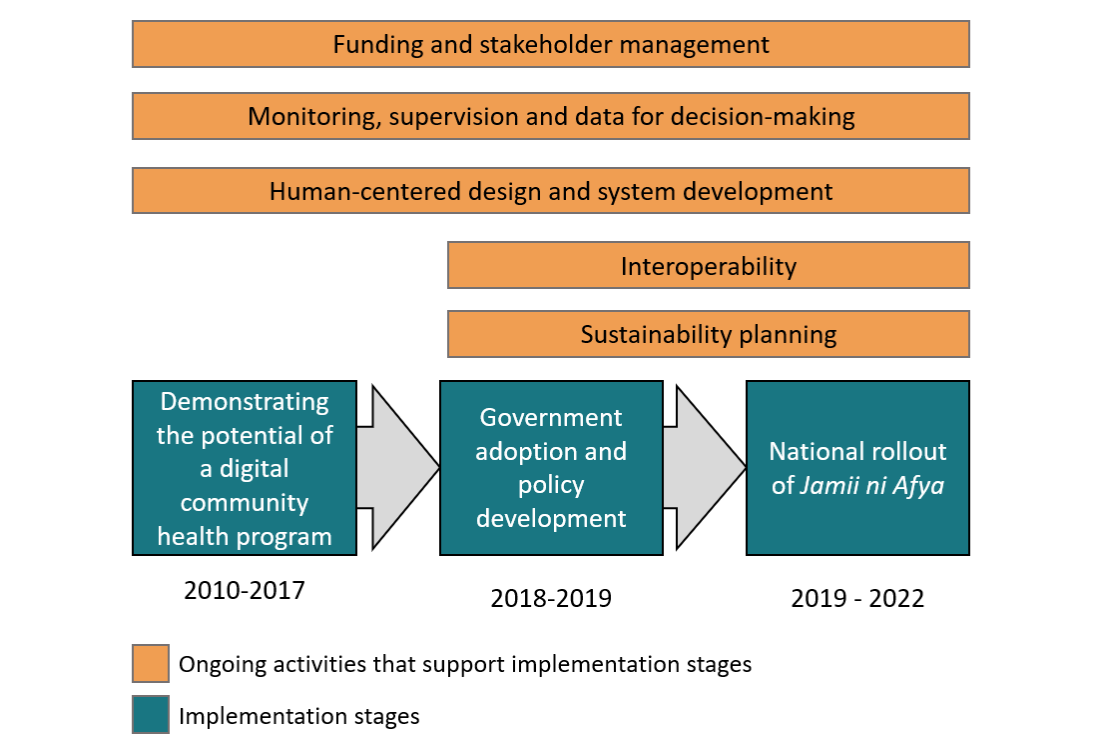
Stages of the evolution of Zanzibar’s community health program.

In this section, we present the stages of the evolution of Zanzibar’s community health program, which are summarized in Figure 1.

### Demonstrating the Potential of a Digital Community Health Program

D-tree International, a digital health NGO, began working with the RGoZ in 2010 on Safer Deliveries—a digitally enabled maternal and newborn community health pilot project aimed at increasing facility deliveries and improving postpartum care. This project has been described elsewhere [16,17]. Briefly, CHWs were trained, supervised, and equipped with a mobile app to enable them to register pregnant women, provide health education, screen them for danger signs and refer them to a health facility, and link them with locally available transportation. From 2012 to 2018, this project supported 400 CHWs to care for over 80,000 pregnant women and their newborns and demonstrated an increase in pregnant women delivering at health facilities from 50% to 75% (a 50% increase), led to increases in facility-based postpartum care visits from 20% to 80%, and increased the completion of community facility referrals from 27% to 90% [16]. Clients reported receiving personalized, responsive, and compassionate care. They were empowered to provide direct feedback about the quality of facility-based services, which district health teams used to monitor and improve service quality. Health facility staff and District Health Management Teams were involved in the program design to ensure that health facilities—especially primary health care facilities—were prepared for increased demand for maternal health services.

### Government Adoption and Policy Development

**Figure 2:**
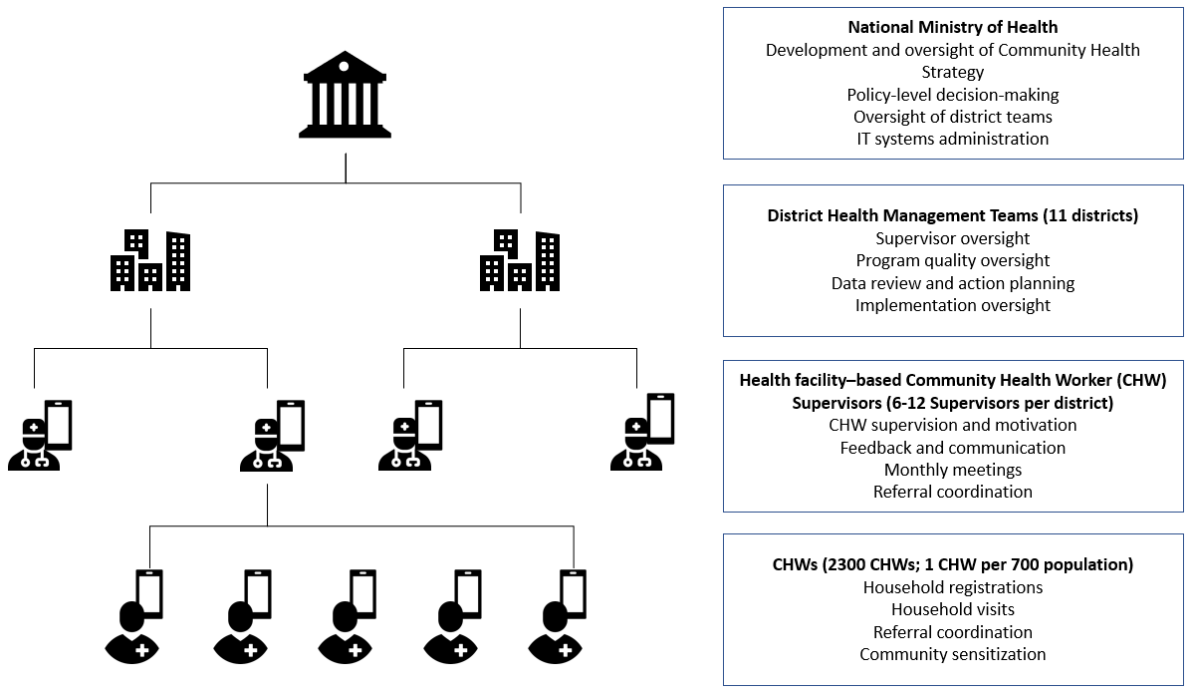
Structure of the Zanzibar community health program. CHW: community health worker.

Based on the success of the Safer Deliveries project and the recognition that a digitally enabled, government-owned community health system was critical to sustainably improve health in general in Zanzibar, the RGoZ adopted this digital CHW program as part of the formal health system at the national level, unifying all community health programs through this single initiative named “Jamii ni Afya” (Swahili for “Community is Health”). In order to formalize this program as a government initiative, the RGoZ updated its community health strategy to make Jamii ni Afya a central pillar of community health. This strategy serves as a formal government document outlining how community health volunteers fit into the larger Zanzibar health system and specifying their roles, responsibilities, training, and required qualifications. Figure 2 summarizes the structure of the community health program. In particular, the revised community health strategy specifies that CHWs will be supported with a digital health platform, as the Ministry of Health Zanzibar recognized the benefits of technology in the pursuit of its vision for a healthy population.

The program uses the term “community health volunteers” to emphasize engagement on a volunteer basis; however, in line with prior literature, we will henceforth refer to them as CHWs.

### Human-Centered Design and Development

From January to June 2019, the Ministry of Health Zanzibar, D-tree International, and other community health stakeholders participated in a human-centered design process to cocreate both the programmatic and technical aspects of the Jamii ni Afya app. The approach was to fully define the health problem and local context first and then select the technology that is best suited to solve that problem. Thus, the program and technology design processes were conducted simultaneously, where program requirements—including health objectives, CHW workflows, supervisory systems, and long-term government management capacity—informed technology design choices. Community health stakeholders included community members, political and religious village leaders, CHWs, CHW supervisors, and District Health Management Teams. Input was initially gathered through sensitization meetings with all abovementioned community stakeholders and then periodically with district managers. Supervisors also had WhatsApp groups to discuss program questions. During app design and development, CHWs were involved in early prototype testing. Further feedback was gathered during CHW training sessions by observing how well CHWs were able to conduct practical exercises on a training app. After implementation, feedback was gathered during field observations. All activities considered the local context, including gathering buy-in from political village leaders; conducting activities with larger groups and broad participation given the communal culture; and understanding basic digital literacy, resulting in involving CHWs in later app design stages.

The Principles for Digital Development [18] were used throughout the design process, specifically “design with the user,” “understand the existing ecosystem,” “design for scale,” and “reuse and improve” to ensure that the resulting system was locally relevant, user-friendly, cost-effective, and sustainable.

**Figure 3:**
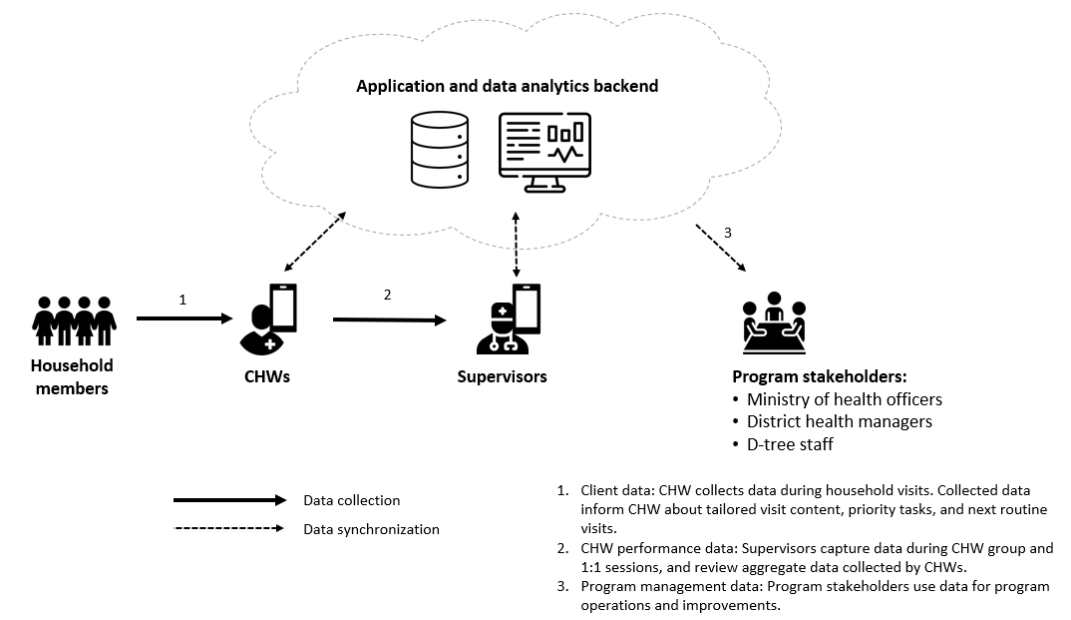
Flow of data within Jamii ni Afya. CHW: community health worker.

Jamii ni Afya leverages government guidelines and global best practices to guide CHWs using digital technology in delivering high-quality health education and counseling services in maternal and child health, nutrition, water, sanitation and hygiene, and early childhood development. The data generated from these interactions are used to further personalize health services by tailoring health messages based on the personal circumstances of the client, improving supervision, and supporting programmatic and policy decision-making at the community, district, and national levels. For example, if a pregnant woman has specific risk factors (based on government guidelines), the app flags that she should plan to deliver at a hospital rather than a primary health facility, and subsequent counseling within the app supports the woman to develop a birth plan for delivery at the nearest hospital. If a CHW indicates that a child has developmental delays (based on observing the child and caregivers at home), the app suggests specific play activities that the caregivers can undertake to support the child’s development. Figure 3 illustrates the data flow for the program.

### Interoperability

The Jamii ni Afya mobile app is built on the Community Health Toolkit (CHT), an open-source global goods platform developed to support community health workers globally. This platform was selected by the Zanzibar government due to the following reasons: it is open-source and uses well-known components and frameworks, it has a growing community that can be leveraged for support, it can be hosted in a local data center, the skills required to configure health worker tools are found among Ministry of Information, Communications and Technology staff, and it is easily available in the local market. In addition, CHT runs on low-end Android smartphones and has offline functionality, which is critical in Zanzibar where network connectivity is not guaranteed. Developers from D-tree International and the Ministry of Health Zanzibar led the mobile app development process, including system requirements and specifications. The system was designed to ensure interoperability with the broader digital health ecosystem in Zanzibar. The CHT is integrated with the government’s health management information system (District Health Information System [DHIS2]) and provides data for the community health information system. Jamii ni Afya is referenced in the RGoZ’s first-ever digital health strategy, which works to coordinate and optimize government resources for digital health in Zanzibar. It is integrated with the Zanzibar Health Interoperability Layer (based on OpenHIM) and can thus integrate with an up and coming electronic medical records system. There are plans to integrate with the future client registry system and a health care worker registry. To date, interoperability has been achieved at the level of data exchange, which is a foundational level focusing on sending and receiving information without interpreting it. Interpretation occurs at the receiving end in accordance with separately defined specifications. For example, when integrating with DHIS2, the submitted data payload was formatted in accordance with DHIS2 requirements, and the meaning of the data payload was agreed on by the involved technical teams. The OpenHIM-based interoperability layer enables higher levels of interoperability, including interoperability of data structures, meaning of data, process, organizational rules, etc. This affords the interpretation of data and information without a system having to understand the DHIS2 or CHT, and makes interoperability with additional systems and platforms easier. However, the technical teams agreed that OpenHIM would initially only pass through payloads—that is, it would not use the higher-level interoperability options—to speed up implementation. For new integrations and for updated integrations, it is planned to leverage terminology and other data standards, which will be consistent with the planned electronic medical record system, but the specific standards are still under discussion.

### National Rollout

Jamii ni Afya was introduced in phases from July 2019 to August 2021 to all of Zanzibar’s 11 districts. Throughout the rollout, the Ministry of Health Zanzibar played a central role, from recruiting and training CHWs and supervisors, raising awareness in communities, and coordinating supportive supervision for health workers. The standard implementation approach used to scale up the project included community sensitization, recruitment and selection of CHWs and health facility–based supervisors, 10 days of training on the overall program and mobile app, and intensive mentorship for a 3-week period, followed by ongoing monthly supervision and semiannual refresher trainings.

### Monitoring, Supervision, and Data-Driven Decision-Making

As a by-product of program implementation, Jamii ni Afya collects extensive data on enrollment, health care usage, and health outcomes. These data are made available on program dashboards that are used by district health management teams to monitor program progress and CHW performance, track health trends, and identify challenges. Supervisors have a mobile app that displays data on the CHWs they supervise, enabling tailored and prompt follow-up and support. We leverage these data in 3 important ways. First, we continuously evaluate the process of program rollout and scale-up to identify successes and areas for improvement [17]. Second, we evaluate the impact of specific components of our program on key outcomes. For example, we found that the number and timing of CHW visits increases the likelihood of health facility delivery [19]. Third, we develop and embed prediction models built using machine learning to tailor our program to individuals and communities [20].

Insights from monitoring and evaluation are used to inform ongoing quality improvement. In 2017, district health managers used the program dashboard to identify that 37% of women were paying for antenatal services, even though they are supposed to be free of charge. The district health team learned that facilities were chronically out of stock for the reagents for tests performed at antenatal visits, and needed to charge clients for services so they could procure these reagents from local pharmacies. With this information, the district health team met with the District Commissioner and presented the data and findings. They were able to obtain a commitment to increase the district health budget to cover the cost of these reagents. Prior to the digital program, district managers knew women were paying for health services but could not back this up with data and were, therefore, never able to advocate for increased funding. Due to this action by the district health team, payments for antenatal care decreased immediately from 37% to 1%.

With Jamii ni Afya established as a fully scaled national government program, there is also increasing interest to leverage the resulting data for other purposes. For example, the RGoZ is exploring how Jamii ni Afya can collect household socioeconomic data during routine visits, which can inform subsidies for the upcoming Universal Health Insurance program.

### Sustainability Planning

RGoZ’s leadership has been critical to Jamii ni Afya’s success and instilled trust in the population for the long-term sustainability of the program. By establishing Jamii ni Afya as part of a national strategy, the government has been able to formally assign oversight and implementation responsibility to government staff so that running the program becomes part of their job description and regular responsibility. The government directly pays the salaries of Jamii ni Afya CHW supervisors (210 individuals), who are health care providers working at health facilities and are 100% dedicated to community health. In addition, 1 person in each of Zanzibar’s 11 districts acts as a District Health Promotion Focal Person for Jamii ni Afya. These government employees are responsible for district-level coordination of the program, which has been critical for program oversight, government commitment, and ownership.

The Zanzibar Community Health Strategy Costed Operational Plan developed by the Ministry of Health Zanzibar in 2022 found that the annual operating cost for Jamii ni Afya is TZS 6.4 billion (approximately US $2.73 million), which is equivalent to US $1.70 per capita. Currently, the government is spending US $0.30 per capita on Jamii ni Afya salaries. The remaining costs are covered by donor funds, which funds equipment and working tools, CHW stipends, training, meetings, technology, and hosting costs. The RGoZ has committed to increasing their funding commitments by 25% each year for the next 4 years, until they are fully funding the program by the end of 2026. Of note, the cost per capita is the cost of the entire community health program, which includes the digital health system. We have not isolated the digital system costs since technology is fully integrated in the community health program and cannot be viewed as a stand-alone cost.

### Funding and Stakeholder Management

Since the program’s inception in 2010, a variety of funders have contributed to the development, piloting, and scale of Jamii ni Afya. From 2010 to 2014, the pilot project was funded by the Bill & Melinda Gates Foundation through their Grand Challenges initiative, which enabled early evidence generation. From 2014 to 2018, the project was funded by the Saving Lives at Birth Grand Challenges Transition to Scale grant, which allowed the program to scale to all districts in Zanzibar and further generate evidence. In 2018, when the program was formally adopted by the RGoZ, funders included Fondation Botnar and the Human Development Innovation Fund. Since 2019, the program also received cofunding from the James Percy Foundation, UNICEF (United Nations Children's Fund), the Conrad N. Hilton Foundation, Google AI for Social Good, the Patrick J. McGovern Foundation, and Jhpiego.

## Implementation (Results)

Since August 2021, Jamii ni Afya has been operating on a full national scale. In total, 2300 CHWs and 210 supervisors have been trained to support every household in Zanzibar. As of March 2023, more than 1.5 million people had been registered to the system, representing nearly 80% of Zanzibar’s population, and over 320,000 pregnant women and children younger than 5 years have received health visits.

Both the current Jamii ni Afya and prior Safer Deliveries programs focus on education and promotion of health-seeking behavior for maternal and child health and have played a role in positive trends in key health outcomes. The percentage of women delivering at a health facility increased from a baseline of 64% [11] in 2015 to 85% among women registered in the program in 2022, implying a 33% increase. In addition, rates of stunting reduced from 30.4% in 2015 to 17.6% in 2022 [11,21]. While we cannot attribute these reductions to Jamii ni Afya, we are confident that the program’s focus on education and promotion of health-seeking behavior for maternal and child health played a role in these significant changes. To directly quantify the Jamii ni Afya’s impact on early childhood development outcomes, our team implemented a nationally representative baseline survey in 2018 with a postimplementation survey planned for late 2023 [22].

There were many factors that led to the success of Jamii ni Afya as a nationally scaled, digital community health program. Briefly, a key element was designing the program by starting with the health challenge and local context rather than the technology. Only when the health challenge was defined and a clear vision for what was possible was conceptualized did we bring in technology, which enabled us to focus on the need, rather than the technology solution. In addition, we leveraged the Principles for Digital Development to guide the technology design process. We also heavily focused on the use of data for decision-making, which amplified the value of the program beyond service delivery, and demonstrated how population-level data could add tremendous value to planning, budgeting, and policy-making at the local, district, and national levels. Finally, extensive collaboration with local and global partners was critical to the success of Jamii ni Afya. The project team engaged with global technology partners, health experts, evaluators, data scientists, and data governance coalitions. Within Zanzibar, collaboration with health experts, universities, political strategists, and communication experts helped to shape the program and support its sustainability. Collaboration with long-term and flexible funders was also key to Jamii ni Afya’s success, enabling the project to adapt on the basis of evolving needs and understanding throughout various stages of maturity.

While there were many success factors, there are also a number of challenges and lessons learned. In general, a key lesson is that scaling and sustaining a national digital health program is time-consuming. When we began implementing in 2010, digital health was not a key priority for the RGoZ. It took years to demonstrate the value of the system and get key leaders on board. In addition, it is challenging to successfully scale a digital health intervention when a country has low maturity in foundational aspects of digital health overall (such as digital health leadership and governance, digital health infrastructure, standards and interoperability, and health workforce capacity in digital health). Over the course of the last 13 years, we have been supporting the RGoZ to advance its digital health capacity, which is critical to sustain the Jamii ni Afya program. However, this takes time and significant investment. In addition, managing competing stakeholder interest is a challenge. Often, funders and implementing organizations push for their own interests to be prioritized, which may not be aligned with government priorities. We have been working closely with the Ministry of Health Zanzibar to develop processes and guidelines to manage stakeholder demands in a way that enables continued investment but also ensures that development of the program is aligned with government interest and need. Finally, government capacity and long-term sustainable financing is a challenge, which we are addressing through a formal transition plan, and describe further in the *Discussion* section.

The current focus on Jamii ni Afya is full transition to the RGoZ. Working with partners, the government has developed a transition plan that outlines the financial, technological, and operational aspects of the program that will be fully absorbed by the government by 2026. D-tree International and partners are supporting skills transfer and systems development to enable this transition, recognizing that financing and management of a nationally scaled digitally enabled community health program is a major undertaking, and strong accompaniment from nongovernmental organizations is critical to the effective handover of such a system.

## Discussion

This implementation report outlines the evolution of Zanzibar’s national, digitally enabled community health program. Since 2010, the program grew from a small pilot to a nationally scaled, government-owned initiative that is set to be fully institutionalized within the national health system. During this time, we have learned many lessons and best practices that may be relevant to governments and practitioners designing, scaling, and institutionalizing digitally enabled community health programs.

Community health programs have traditionally been driven by vertical health programs (ie, HIV/AIDS, family planning, or maternal health) and focus on specific health service delivery [9,23]. With Jamii ni Afya, we focused on designing a strong community health system, independent of the types of services being delivered, which serves as a strong foundation on which to add service delivery areas. The Zanzibar Community Health Strategy is a critical document that not only formalizes Jamii ni Afya as a government initiative, but also outlines critical systems-level criteria to guide the program. This includes defining the supervision structure, selection criteria, and training and stipend payments, which is aligned with the World Health Organization’s guidelines for community health [10].

Just as it is important to invest in building strong foundations in the community health system, it is critical to design digital health initiatives that are integrated and harmonized with the broader digital health ecosystem. A recent article by Karamagi et al [24] describes the alarming lack of coordination, integration, scalability, and sustainability of digital health interventions in sub-Saharan Africa. Through the design of Jamii ni Afya, we were intentional in ensuring that the digital system was interoperable with other digital health systems such as DHIS2 in order to enable the transfer of data between systems. The Jamii ni Afya team was also closely involved in the development of the Zanzibar Digital Health Strategy, which features Jamii ni Afya as a core digital health intervention. Jamii ni Afya was among the first systems to adopt the Zanzibar Health Interoperability Layer, which lays the foundation for seamless data exchange across systems. These intentional design decisions, coupled with integrating the program into government policy, have set the stage to ensure long-term coordination and synergy with the evolving digital health landscape in Zanzibar.

Having the right leadership is recognized as a critical lever to accelerate the institutionalization of a product or service [25]. Strong leadership, ranging from the highest levels of government to local community leaders, may be the single-most important success factor for Jamii ni Afya. Extensive work was carried out early on to establish buy-in and trust at the community level. Community sensitization was built into the national rollout plan, leveraging shehas (ward leaders) to champion the initiative within their communities. Sensitization activities were led by district- and national-level government staff who introduced the program as a government-led initiative, and CHWs have official government-issued identification cards that provide increased credibility. These initiatives were critical in building public trust and ownership of Jamii ni Afya from the beginning, resulting in 90% of the population being registered in the program. We also focused on high-level political support to champion this initiative and set the stage for full government ownership and sustainable financing. In February 2023, the Minister of Health Zanzibar presented Jamii ni Afya to the Zanzibar President who pledged his commitment to champion the institutionalization of this initiative. While this support is encouraging, one lesson learned is the importance of high-level political engagement early on. The Jamii ni Afya team initially focused on community, district, and departmental support within the Ministry of Health Zanzibar in order to build buy-in and support, and only after the program was established, began lobbying higher levels of government for their support. Earlier engagement with these higher levels may have expedited the institutionalization process.

The evolution of Jamii ni Afya has lasted nearly 13 years and is now in a strong position to be institutionalized within Zanzibar’s health system. Each stage of the project—beginning with a small pilot to demonstrate the potential of this model, gaining consensus for government adoption, and integrating the program into government policies—was critical to build demand and formalize Jamii ni Afya as a government initiative. Often, donors expect that health systems strengthening initiative, including digital health, should be conceptualized, piloted, scaled, and institutionalized within a short period of a few years. However, our experience is consistent with that of other studies and shows that significant digital health systems transformations take time and require funding and partnerships to accompany governments on their journey to scale [26].

One of the most significant challenges for Jamii ni Afya’s institutionalization is sustainable financing, which is a common challenge for community health programs across many low- and middle-income countries [27]. The RGoZ has committed to full institutionalization of Jamii ni Afya by 2026, which is an ambitious target and will require substantial political commitment and innovative solutions. The Jamii ni Afya program’s team is currently working on a number of strategies to support the government to fully finance the program within 4 years. One way is to increase demand for the program outside of the health sector. Jamii ni Afya champions are in discussion with the Ministry of Finance Zanzibar, Ministry of Social Welfare, Ministry of Agriculture, and Ministry of Education to discuss how existing or new data from Jamii ni Afya could be valuable to them, or the potential of expanding the package of services delivered by CHWs. By extending the value of the program outside of the Ministry of Health Zanzibar, additional government entities can contribute toward operating costs, thus reducing the burden on any one particular ministry and reducing duplication of efforts. We are also in the process of integrating Jamii ni Afya into the government’s upcoming Universal Health Insurance scheme, which will be rolled out in the next 1-2 years. The Jamii ni Afya digital platform can be leveraged to collect data on household economic status and feedback on health facility quality, and CHWs can support registration of households into the program. In turn, the community health program can be partially financed by revenue generated through the Universal Health Insurance scheme. It has been reported that many national health insurance schemes fail to adequately engage the informal sector [28]. Leveraging CHWs to support these efforts in Zanzibar could both increase participation in the informal sector and support operational costs for the community health program. There is also increasing support from multilateral donors who are increasingly investing in digitally enabled community health systems and provide funding directly to governments to decrease financial reliance on nongovernmental partners. This multipronged financing approach is aligned with a recent review that found that an adaptive mix of health financing mechanisms is necessary for low- and middle-income countries [29].

In conclusion, Jamii ni Afya represents one of the world’s first examples of a nationally scaled digitally enabled community health program. This implementation report outlines the evolution of this program, from the pilot to the national scale and institutionalization. While Zanzibar has a relatively small population and is geographically isolated, the challenges they face are similar to many other settings. Indeed, based on the experience in Zanzibar, D-tree International is replicating this approach by supporting government-led digital community health initiatives in Mainland Tanzania and Zambia. However, drawing on work from Greenhalgh et al [30], we also recognize that the spread and scale-up of innovations is a combination of technical, ecological, and social aspects and require social science approaches to understand and facilitate change based on local contextual factors and incentives [30]. The authors hope that the experiences and lessons learned described in this implementation report will be helpful to others working to implement digitally enabled community health systems at scale. In addition, given the maturity of Jamii ni Afya, the relatively small size of Zanzibar’s population, and the significant health need, there is an opportunity for Zanzibar to become a digital health implementation research hub in order to test new and promising innovations, research various implementation models, and develop best practices that can be applicable in other settings.

## Abbreviations

CHT: Community Health Toolkit
CHW: community health worker
DHIS2: District Health Information System
iCHECK-DH: Guidelines and Checklist for the Reporting on Digital Health Implementations
NGO: nongovernmental organization
RGoZ: Revolutionary Government of Zanzibar
UHC: universal health coverage
UNICEF: United Nations Children's Fund
